# Prevalence of Non-insulin-dependent Diabetes Mellitus Among Patients with Cholelithiasis: A Single-centered, Cross-sectional Study

**DOI:** 10.7759/cureus.2444

**Published:** 2018-04-07

**Authors:** Sidra Ali, Shaik Tanveer Ahamad, Abdul Subhan Talpur, Shreeya Parajuli, Jawad Farooq

**Affiliations:** 1 Medicine, Dow University of Health Sciences (DUHS), Karachi, Pakistan; 2 Department of Medicine, Deccan College of Medical Sciences; 3 Medicine, Liaquat University of Medical and Health Sciences, Jamshoro, PAK; 4 Research, Annapurna Neurological Institute and Allied Sciences (anias)

**Keywords:** diabetic complication, gallstones, cholelithiasis, pakistan, cholecystectomy

## Abstract

Introduction

Gallstone disease (GD) is one of the major causes of morbidity and mortality in the west and most of the countries worldwide. Cholelithiasis and diseases of the biliary tract are becoming more prevalent with the socioeconomic burden in developing countries like Pakistan. GD is a chronic, recurrent hepatobiliary disease, the basis of which is the impaired metabolism of cholesterol, bilirubin, and bile acids, which is characterized by the formation of gallstones in the hepatic bile duct, common bile duct, or gallbladder. Epidemiologic studies have shown that individuals with diabetes have a higher risk of cholelithiasis but only a few studies have been done in Pakistan to establish the association so far. Hence, the aim of the present study is to establish the association between diabetes and gallstone disease.

Methods

A cross-sectional study was conducted at Liaquat University Civil Hospital, Hyderabad, Pakistan, between February 2017 and August 2017. Patients between the ages of 10 and 70 from either sex, who were diagnosed with cholelithiasis were included in this study whereas those patients who underwent cholecystectomy previously were excluded. Diabetic cases were identified based on fasting glucose levels (FGL) and the serum levels of HbA1c. An interview-based questionnaire was employed to collect the patient's demographic profile and risk factors by the students. Informed consent was taken from all the study subjects and the confidentiality of the data was ensured.

Results

From the sample size of patients evaluated (a total of 204), based on investigative studies performed, 74 cholelithiatic patients (36.6%) were found to concurrently have diabetes as well. Among the 74 patients with both cholelithiasis and diabetes type-2 (NIDDM), 56 were female and 18 were males. The rest of the patients with cholelithiasis were found to be non-diabetic (78 were males and 52 female). The majority of the GD patients (51 (25 males and 26 females)) in the study sample was in the 50-60 age group. The mean age of the patients was 43 ± 12.1. In this study, we measured the fasting glucose levels (FGL). According to World Health Organization (WHO) and American Diabetes Association (ADA) criteria, we categorized 85 of the GD patients to be non-diabetic with serum fasting glucose levels between 70 and 100 gm/dL, and 45 patients were categorized to be in the pre-diabetic group with FGL levels between 100 and 126. Out of the 204 samples with GD, we found that 74 patients have diabetes, with serum FGL >126mg/dL. We measured HbA1c from each individual in the study sample. It was found that 79 patients had HbA1c levels <5.5, they are categorised as non-diabetic according to WHO and ADA criteria, 51 patients had values between 5.5 and 6.5 (pre-diabetic), and 35 GD patients had HbA1c values between 6.5 and 7.5 (categorized as diabetics with good control) and 39 patients with HbA1c above 7.5 (diabetes with poor control).

Conclusion

In this study, we concluded that there is a higher prevalence of NIDDM in GD patients and there is an association between GD and NIDDM. This study also reiterated the association between obesity and GD. Female sex and advancing age also contribute to the formation of cholelethiasis. Cigarette smoking and alcohol consumption further worsen cholelithiasis but are not established primary risk factors.

## Introduction

Gallstone disease (GD) constitutes a significant health problem in developed societies, affecting 10% to 15% of the adult population [1]. It is estimated that 7% to 10% of the world’s population has either symptomatic or asymptomatic cholelithiasis [2]. The prevalence of diabetes mellitus in Pakistan is estimated to be around 11.77% [3]. Individuals with diabetes are at a higher risk of developing gallstones and acalculous gallbladder disease apart from gallstones. Cholelithiasis may progress more rapidly in patients with diabetes, which is due to chronic and severe infections of the gallbladder in diabetics. Globally, the reported prevalence of gallstone disease in diabetes mellitus is 36.2% [4]. The cholelithiasis tends to occur predominantly among multiparous, obese women and in those who use combined oral contraceptives (OCPs). In postmenopausal women, hormone replacement therapy (HRT) increases the risk for cholelithiasis significantly due to its estrogen component, which is shown to cause biliary stasis [5]. It's incidence increases with age [6-7] and parity [7].

One of the risk factors for gallstone disease (GD) is diabetes mellitus type 2. It is related to the metabolic abnormalities associated with overweight, obesity, insulin resistance, dyslipidemia, and dietary habits [8-9]. Epidemiologic studies have shown that individuals with diabetes have a higher risk of cholelithiasis, although not universally accepted [10]. Likewise, there is no consensus on what is the most appropriate course of action for diabetic patients with gallstones. The literature reports a higher incidence of gallstone disease in diabetic patients; this fact may be related to the dietary habits of individuals with diabetes [11]. However, autonomic neuropathy is considered to be responsible for the lithiasis tendency in this group of patients [12]. In a study that included 566 cholecystectomies performed for acute cholecystitis, 123 of the patients were diabetic and 433 were non-diabetic but showed a considerably higher morbidity (21% vs. 9%) and mortality rates [13]. In a study where 72 emergency cholecystectomies done for GD along with the control group (age- and sex-matched), diabetic patients had more complications (38.9%) than non-diabetics (20.8%). Infections in this patient population are three folds higher and the primary reason in GD cases to cause complications. Validating these findings, a study conducted on the autopsies of patients with diabetes showed that although rare, severe complications and death from cholelithiasis were significantly more common in diabetic patients than in non-diabetics [14].

The susceptibility to infections in diabetic patients leads to fatal complications since high serum glucose levels, hyperinsulinemia, dehydration, malnutrition, and vascular disease collectively lead to the dysregulation of host immune responses, eventually resulting in defective and weak neutrophilic phagocytosis, chemotaxis, and intracellular bactericidal activity [15]. Risk factors for cholelithiasis include age, hypertriglyceridemia, genetic predisposition, drugs (clofibrate, OCPs, and ceftriaxone), terminal ileal resection and gallbladder hypo-motility as seen in post-vagotomy, and total parenteral nutrition (TPN). Ultrasonography (USG) remains the primary diagnostic modality for imaging the biliary system and is particularly useful for examining the gallbladder. A gallstone appears as an echogenic structure within the gallbladder lumen that casts a distal acoustic shadow. USG is sensitive for the diagnosis of gallstones in up to 96% of cases [16].

There are three kinds of pathogenic abnormalities believed to be the cause of cholesterol gallstone formation: super-saturation of bile in cholesterol, increased nucleation of cholesterol crystals, impaired gallbladder emptying with stasis, and decreased motility of the intestine. Cholesterol gallstones are made mainly of cholesterol crystals, which are formed due to a defective cholesterol metabolism. Diabetes is a chronic metabolic condition wherein the tissues either do not produce enough Insulin (insulin-independent DM/type 1) or do not effectively respond to insulin due to peripheral insulin resistance (insulin-dependent DM/type 2). This results in glucose (an osmotic hexose sugar) increasing in the blood (hyperglycemia), leading to several potential complications. The relationship between blood cholesterol, low-density lipoprotein (LDL), and high-density lipoprotein (HDL) levels and cholesterol gallstone formation is complex and multifarious.The prevalence of diabetes mellitus in Pakistan is estimated to be 11.3% [17]. Individuals with diabetes are at a higher risk for gallstones and acalculous gallbladder disease (apart from GD). Gallbladder disease may progress more rapidly in patients with diabetes, who tend to suffer more severe infections as compared to the general population. The reported prevalence of gallstone in diabetes mellitus is 36.2% [4].

Considering the fact that there were only a few previous studies been conducted on the association between diabetes in GD in Pakistan. The present study was being carried out at Liaquat University Hospital, which is a tertiary care hospital in Hyderabad. The study primarily intended to focus on the prevalence of NIDDM among cholelithiasis patients in the Pakistani population.

## Materials and methods

A cross-sectional study was carried out at Liaquat University Hospital (LUH), Hyderabad, Sindh, between February 2017 and August 2017. The study was approved by the ethics committee of Liaquat University of Medical and Health Sciences. The study population encompassed 204 patients between 10 and 70 years of age from both sexes who were diagnosed as a case of cholelithiasis during the study period. The patients who underwent cholecystectomy previously and patients under the age of 10 were excluded. An interview-based questionnaire was designed by authors employed to collect the patient's demographic profile and risk factors. The questionnaire started with specific instructions followed by demographic data. Doctors of LUH commented on the relevance and unambiguity of the questionnaire. With their suggestions and opinions, the necessary modifications were done and the questionnaire was validated with Alpha Cronbach 0.69.

The diagnosis of diabetes was made according to American Diabetes Association guidelines [18]. Height was measured by a non-stretchable plastic measuring tape after making the subject to stand straight against an even wall. The body weight of all the subjects was measured by using a standardized weighing machine, which was calibrated in kilograms. Body mass index (BMI) was calculated as weight in kg/height in square meter. Waist circumference was measured using a non-stretchable plastic measuring tape over the unclothed abdomen, at the umbilical level, in the standing position. Hip circumference was measured over light clothing at the widest point over the buttocks when viewed from the side. Waist-to-hip ratio (WHR) was obtained by dividing the waist circumference by hip circumference. Data were entered and analyzed using the Statistical Package for Social Sciences (SPSS), version 23.0 (IBM Corp., Armonk, NY). Frequency and percentage were used to interpret results. Informed consent was taken from all the subjects and the confidentiality of the data was ensured. Participants were informed about the estimated duration of the interview and voluntary participation was emphasized. The interviews were not recorded and participants did not receive any kind of compensation. We excluded the cases that had already undergone cholecystectomy or any other surgery.

## Results

**Figure 1:**
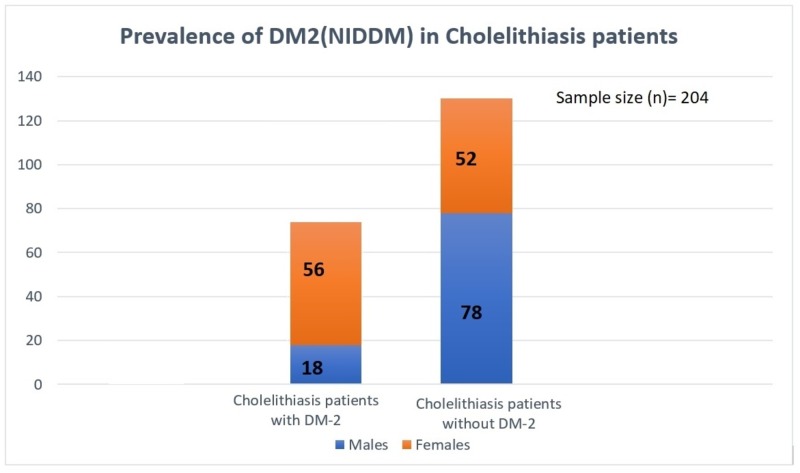
Histogram Depicting the Prevalence of GD in Diabetics vs Non-Diabetics NIDDM: non-insulin-dependent diabetes mellitus; GD: gallstone disease

Out of the total sample size of patients with a diagnosis of GD, 74 patients (36.3%) were found to have diabetes. Among those, 56 patients were females and 18 patients were males. The rest of the patients with cholelithiasis were found to be non-diabetic, as illustrated in Figure 1.

**Figure 2:**
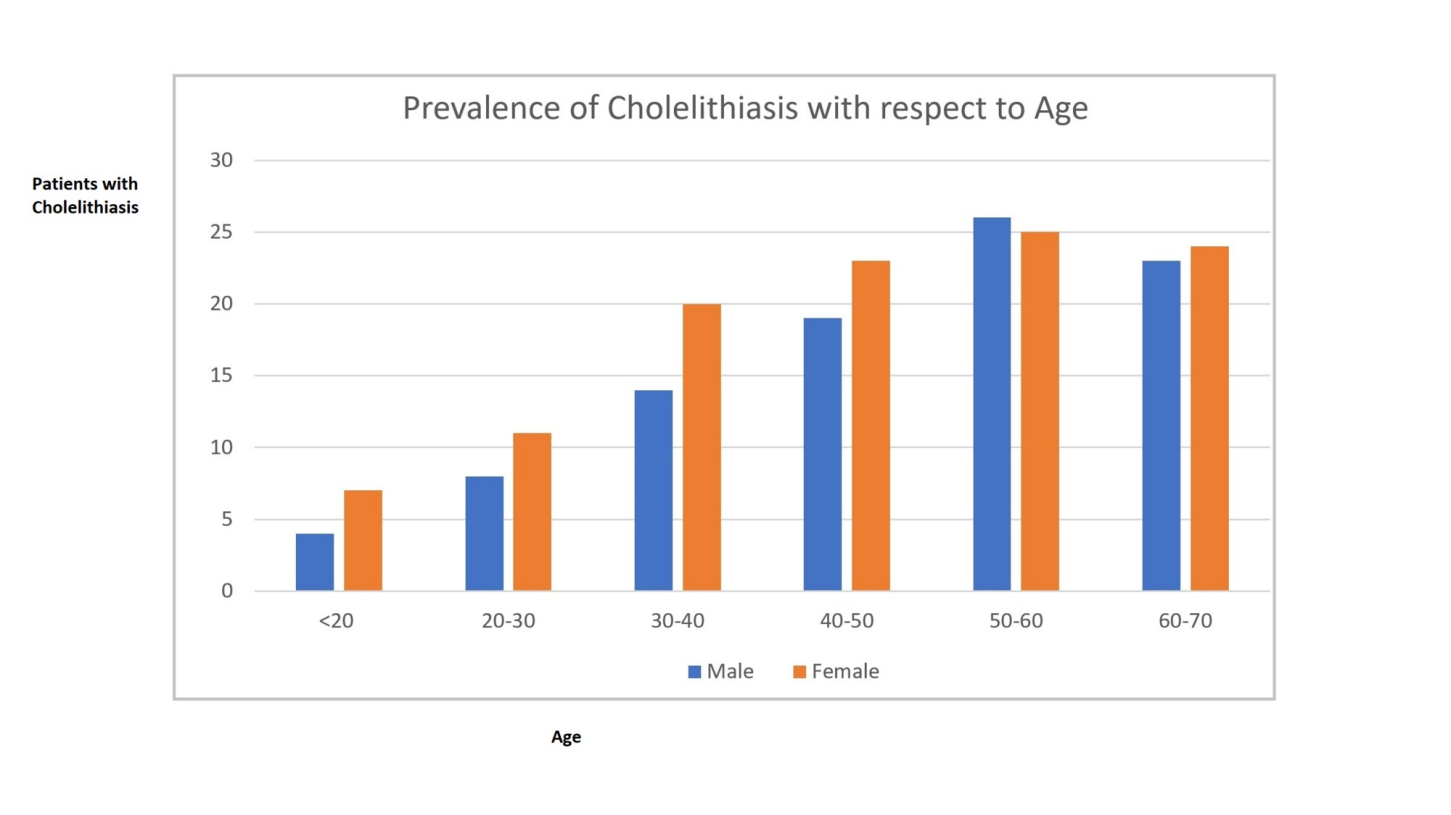
Prevalence of Cholelithiasis with Respect to Age

The mean age of the patients reported was 43 ± 12.1. Among the total cases of GD, there were 11 cases under the age of 20 (four males and seven females), 19 cases (eight males and 11 females) between the ages of 20 and 30, 34 cases (14 males and 20 females) between the ages of 30 and 40, 42 cases (19 males and 23 females) between the ages of 40 and 50, and 47 cases (23 males and 24 females) in the age group of 60-70. The majority of GD patients were in the age group of 50-60, as illustrated in Figure 2.

Among the total cases, we found that 8 (3.9%) were underweight (BMI<18.5), 96 (47%) were overweight (23.1-27.5), and 36 (17.6%) were obese (BMI over 27.5), as shown in Table 1. 

**Table 1:** Demographic Profile of the Study Population BMI: Basal Metabolic Rate

Variable	Value n (%)
Age	43 ± 12.1
Sex a) Male b) Female	94 (46.3) 110 (53.7)
BMI a) <18.5 b) 18.5-23 c) 23.1-27.5 d) >27.5	8 (3.9) 65 (31.8) 96 (47) 36 (17.6)

According to ADA criteria, we categorized 85 GD patients as non-diabetic, 45 patients as pre-diabetic, and 74 patients as diabetic (Table 2). As per HbA1c, 35 had diabetes with good control and 39 had diabetes with poor control (Table 3).

**Table 2:** Fasting Serum Glucose levels in Cholelithiasis Patients (n=204)

Fasting Serum Glucose Levels	Number of Cholelithiasis Patients
Normal (70-100 gm/dL)	85
Pre-diabetes (100-126 gm/dL)	45
Overt Diabetes (>126 gm/dL)	74

**Table 3:** Serum HbA1c Levels in the Study Sample HbA1c: hemoglobin A1c

HbA1c	Number of Cholelithiasis Patients
Non-diabetic (<5.5)	79
Pre-diabetes (5.5-6.5)	51
Diabetes with good control (6.5-7.5)	35
Diabetes with poor control (>7.5)	39

Along with the fasting glucose level (FGL) and hemoglobin A1c (HbA1c) of the study sample, the serum lipid profile was also calculated. The values of each lipid parameter are given in Table 4.

**Table 4:** Serum Lipid Profile Among the Sample Cholelithiasis Patients

Serum Parameter	Sample Size (n=204) with Range		
	Desirable	Borderline	High Risk
Cholesterol	73	95	36
HDL	67	122	15
LDL	82	103	19
TGs	85	108	11

## Discussion

Gallstone disease (cholelithiasis) is one of the most common gastrointestinal diseases, which occurs within the biliary tree, including the gallbladder and the common bile duct. Several studies across the world reported an increased prevalence of gallstones in patients with diabetes mellitus [1]. The primary aim of this study was to assess the association and prevalence of diabetes mellitus (NIDDM) among cholelithiasis patients in Pakistan. In this study, the prevalence of diabetes among GD patients was noted to be 36.3%. However, it was 14.3% in the study conducted by Méndez-Sánchez et al. [19]. In a similar, Swedish study, the prevalence was 15% [20].

Ultrasonography (USG) has played a major role in the diagnostic protocol. It is a relatively risk-free and cost-effective diagnostic modality and provides for the screening of large populations. Evidently, the prevalence varies across the ethnicities, and there appear to be higher rates of cholelithiasis in western Caucasian, Hispanic, and Native American populations and lower rates in Eastern European, African American, and Japanese populations [20-21]. Similarly, Sodhi JS et al. noted a 17.7% prevalence of cholelithiasis in patients with type 2 DM in their study [3].

Munir Jalil Abdul has established that the frequency of gallstones is more in diabetic patients (28%) as compared to those in non-diabetics (12%) in his study.These results were in coherence with a previous study that was done by AL-Bayatiet et al. who showed that there was a higher prevalence of gallstone in diabetics (33%) as compared to controls (17%). Also, El Mehdawi R et al. [22], showed that 39.75% of type 2 diabetes mellitus patients have ultrasonographic (USG) evidence of gallstones as compared to 17.5% of healthy subjects. Obesity is an established risk factor for the development of gallstones. This study has shown that the gallstones were more prevalent in obese diabetics than in non-diabetics at 30.1% and 19%, respectively.These results are in coherence with a study done by A B Olokoba et al. [23], who concluded that diabetic patients had a significantly higher body mass index (BMI) than the controls (P <0.05) with 100 type 2 diabetic patients and 100 age- and sex-matched controls. However, obesity alone is an independent risk factor for obese type 2 diabetic patients and a number of other factors contribute to the increasing incidence of gallstones in type 2 diabetes mellitus. The prevalence and association between diabetes mellitus (NIDDM) and gallstone disease (GD) are based on epidemiological studies. GD has been found to be more prevalent in people with diabetes [23-24], and diabetes is more commonly seen in patients with cholelithiasis [25-26].

Benefits

This cross-sectional study was a feasible, cost-effective, and convenient method to establish the prevalence of NIDDM in GD and identify the association between them. Participants from either gender and of different chronological ages were observed and compared.

Limitations

The limitations of the study include a small sample size, the inclusion of one study site, and confounding factors. The family stress and coping questionnaire (FSCQ) and support questionnaires used in this study have good internal consistency but are not validated. Further studies are needed with multiple sites across the country encompassing both rural and urban settings with a larger sample size.

## Conclusions

In this study, we concluded that there is a higher prevalence of NIDDM in GD patients and identified an association between GD and NIDDM. This study also reiterated the association between obesity and GD. Female sex and advancing age also contribute to the formation of cholelethiasis. Cigarette smoking and alcohol consumption further worsens cholelithiasis but are not established primary risk factors.

## References

[1] Shaffer EA (2005). Epidemiology and risk factors for gallstone disease: has the paradigm changed in the 21st century?. Curr Gastroenterol Rep.

[2] Guimarães S, Gomes H, de Oliveira C (2016). Prevalence of cholelithiasis in patients with type 2 diabetes and obesity in a basic family health centre in Irecê, Northeastern Brazil. Open J Endocr Metab Dis.

[3] Meo SA, Zia I, Bukhari IA ( 2016). Type 2 diabetes mellitus in Pakistan: current prevalence and future forecast. JPMA.

[4] Chapman BA, Wilson IR, Frampton CM, Chisholm RJ, Stewart NR, Eagar GM, Allan RB (1996). Prevalence of gallbladder disease in diabetes mellitus. Dig Dis Sci.

[5] Coelho JC, Bonilha R, Pitaki SA (1999). Prevalence of gallstones in a Brazilian population. Int Surg.

[6] Roesch-Dietlen F, Pérez-Morales A, Melo-Santisteban G, Díaz-Blanco F, Martínez-Fernández S, Martínez JA, Cid-Juárez S (2008). Frequency and clinical, biochemical and histological characteristics of nonalcoholic fatty liver disease in patients with gallstone disease [Article in Spanish]. Cir Cir.

[7] Barbara L, Sama C, Labate AM (1987). A population study on the prevalence of gallstone disease: the sirmione study. Hepatology.

[8] Torres OJ, Barbosa ÉS, Pantoja PB, DinizIII MCS, Sousa da Silva JR, Czeczko NG (2005). Sonographic prevalence of cholelitiasis in out-patients. Rev Col Bras Cir.

[9] Salim MT, Cutait R (2008). Videolaparoscopy complications in the management of biliary diseases. ABCD.

[10] Elmehdawi RR, Elmajberi SJ, Behieh A, Elramli A (2008). Prevalence of gall bladder stones among type 2 diabetic patients in Benghazi Libya: a case-control study. Libyan J Med.

[11] Sami W, Ansari Ansari, T T, Butt NS, Hamid MRA (2017). Effect of diet on type 2 diabetes mellitus: a review. Int J Health Sci (Qassim).

[12] Ferreira AC, Mauad Filho F, Mauad FM, GadelhaI A, SparaI P, Filho IJ (2004). Fatores de risco clínicos e ultra-sonográficos relacionados à litíase vesicular assintomática em mulheres [Article in French]. Radiol Bras.

[13] Souza LJ, Chalita FE, Reis AF (2003). Prevalência de diabetes mellitus e fatores de risco em Campos dos Goytacazes, RJ [Article in French]. Arq Bras Endocrinol Metabol.

[14] Mantovani M, Leal RF, Fontelles MJ (2001). Incidência de colelitíase em necropsias realizadas em hospital universitário no município de Campinas-SP [Article in French]. Rev Col Bras Cir.

[15] Zironi G, Modolon C, Cavazza M (2007). Emergency ultrasound and gallstone ileus. Eur J Emerg Med.

[16] Shera AS, Jawad F, Maqsood A (2007). Prevalence of diabetes in Pakistan. Diabetes Res Clin.

[17] Sodhi JS, Zargar SA, Khateeb S (2014). Prevalence of gallstone disease in patients with type 2 diabetes and the risk factors in North Indian population: a case control study. Indian J Gastroenterol.

[18] Méndez-Sánchez N, Jessurun J, Ponciano-Rodríguez G, Alonso-De-Ruiz P, Uribe M, Hernández-Avila M (1993). Prevalence of gallstone disease in Mexico. Dig Dis Sci.

[19] Muhrbeck O, Ahlberg J (1995). Prevalence of gallstone disease in a Swedish population. Scand. J Gastroenterol.

[20] American Diabetes Association (2017). Classification and diagnosis of diabetes. Sec. 2. In Standards of Medical Care in Diabetes—2017. Diabetes Care.

[21] Sandler RS, Everhart JE, Donowitz M (2002). The burden of selected digestive diseases in the United States. Gastroenterology.

[22] Sichieri R, Evertiart JE, Roth HP (1990). Low incidence of hospitalization with gallbladder disease among blacks in the United States. American journal of epidemiology. Am J Epidemiol.

[23] Olokoba AB, Bojuwoye BJ, Katibi IA, Salami K, Olokoba LB, Braimoh KT, Inikori AK (2006). The effect of type 2 diabetes mellitus on fasting gallbladder volume. African Sci.

[24] Saxena R, Sharma S, Dubey DC (2005). Gallbladder disorder in type 2 diabetes mellitus cases. J Hum Ecol.

[25] De Santis A, Attili AF, Corradini SG (1997). Gallstones and diabetes: a case‐control study in a free‐living population sample. Hepatology.

[26] Jørgensen T (1989). Gall stones in a Danish population. Relation to weight, physical activity, smoking, coffee consumption, and diabetes mellitus. Gut.

